# Has the shortage of fludarabine altered the current paradigm of lymphodepletion in favor of bendamustine?

**DOI:** 10.3389/fimmu.2023.1329850

**Published:** 2023-11-22

**Authors:** Dimitrios Filioglou, Muhammad Husnain, Sharad Khurana, Richard J. Simpson, Emmanuel Katsanis

**Affiliations:** ^1^ Department of Pediatrics, University of Arizona, Tucson, AZ, United States; ^2^ Department of Medicine, University of Arizona, Tucson, AZ, United States; ^3^ The University of Arizona Cancer Center, Tucson, AZ, United States; ^4^ Department of Immunobiology, University of Arizona, Tucson, AZ, United States; ^5^ School of Nutritional Sciences and Wellness, University of Arizona, Tucson, AZ, United States; ^6^ Department of Pathology, University of Arizona, Tucson, AZ, United States

**Keywords:** fludarabine, cyclophosphamide, bendamustine, lymphodepletion, CAR (chimeric antigen receptor) T cells

## Abstract

The most common lymphodepletion regimen used prior to infusion of chimeric antigen receptor-T cells (CAR-T) is cyclophosphamide (CY) in combination with fludarabine (Flu) (CY-FLU). While cyclophosphamide (CY) possesses lymphotoxic effects, it concurrently preserves regulatory T cell activity, potentially affecting the efficacy of CAR-T cells. Moreover, the use of fludarabine (FLU) has been linked to neurotoxicity, which could complicate the early detection of immune effector cell-associated neurotoxicity syndrome (ICANS) observed in CAR-T cell therapy. Given the ongoing shortage of FLU, alternative lymphodepleting agents have become necessary. To date, only a limited number of studies have directly compared different lymphodepleting regimens, and most of these comparisons have been retrospective in nature. Herein, we review the current literature on lymphodepletion preceding CAR-T cell therapies for lymphoid hematologic malignancies, with a specific focus on the use of bendamustine (BEN). Recent evidence suggests that administering BEN before CAR-T cell infusion yields comparable efficacy, possibly with a more favorable toxicity profile when compared to CY-FLU. This warrants further investigation through randomized prospective studies.

## Introduction

Chimeric antigen receptor-T cell (CAR-T) therapy has demonstrated remarkable efficacy in treating relapsed/refractory B-cell malignancies, including but not limited to diffuse large B-cell lymphoma (DLBCL) and B-cell acute lymphoblastic leukemia (B-ALL) (1). More recently the development of anti–B-cell maturation antigen (BCMA) CAR-T cell therapy, has shown promise in patients with multiple myeloma (MM) (2). Lymphodepletion is consistently applied prior to infusion of CAR-T cells to facilitate their expansion and persistence (3). The most common lymphodepleting regimen for commercially approved CD19-CAR-T cell therapy in young adults and children has been cyclophosphamide (CY) (500 mg/m^2^× 2 days) and fludarabine (FLU) (30 mg/m^2^ × 4 days) (CY-FLU) (4). Although several dosage alterations have been tested, the selection of this lymphodepletion approach has not undergone prospective investigation, raising the question of whether there may be more advantageous alternatives (5–7). Due to the extended shortage of FLU in the United States, numerous institutions, including ours, have turned to Bendamustine (BEN) as a viable alternative to CY-FLU, with promising results (8, 9).

## Effects of cyclophosphamide on immune function: immunomodulatory, immunosuppressive, and lymphodepletion aspects

CY is an inactive prodrug that requires enzymatic and chemical activation by CYP450 enzymes in the liver. The resultant nitrogen mustard promotes the interstrand and intrastrand DNA crosslinking that account for its cytotoxic properties (10). CY is associated with a range of common adverse effects, including bone marrow suppression, gonadal toxicity, and carcinogenesis. Additionally, it can lead to more distinct complications such as hemorrhagic cystitis, cardiomyopathy and interstitial lung disease (10). CY is deactivated mainly by the isoform 1 of cellular aldehyde dehydrogenase (ALDH1), which is highly expressed in cells with high proliferative potential such as hematopoietic stem cells. Significantly, ALDH1 is minimally expressed in lymphocytes which provides a rationale for the use of CY as a lymphodepleting agent (11). Saida et al, investigated the use of CY in lymphodepletion and found that it eradicated lymphocytes efficiently; however, the percentage of regulatory T cells (Tregs) was significantly increased (12). Terao et al. indicated that an early rise in Tregs following Tisagenlecleucel infusion is linked to a reduced risk of cytokine release syndrome (CRS) and could potentially serve as a predictive marker. Nevertheless, despite this Treg increase, there was no discernible improvement in the overall survival (OS) of these patients (13). Indeed, it is widely recognized that Tregs suppress anti-tumor responses and have the capacity to inhibit CAR-T cell activity (14). As such, one of the intended advantages of lymphodepletion is the simultaneous reduction of all lymphocytes including Tregs (15–20). CY given following hematopoietic cell transplantation (PT-CY) preserved Tregs during the early immune reconstitution period (21). The same group also reported that PT-CY led to rapid recovery of CD4^+^Foxp3^+^ T regulatory cells, which were later found to be resistant to CY via upregulation of ALDH (22). Similarly, in murine hematopoietic cell transplant models they confirmed that PT-CY preferentially recovered Tregs at day +21 while restraining the proliferation and the differentiation of alloreactive CD4^+^ conventional T-cells (23). While these effects ameliorate graft versus host disease (GvHD), they may simultaneously reduce the efficacy of graft versus leukemia (GvL) responses. In addition to its effects on lymphoid populations CY also influences myeloid subsets including myeloid derived suppressor cells (MDSCs), which are highly immunosuppressive and impeding their effects may improve the efficacy of adoptive immunotherapy (24). Mechanistically, MDSCs reduce the levels of l-arginine and l-tryptophan by expressing arginase-I and indoleamine 2,3-dioxygenase (IDO), thereby suppressing T cell function (25). Interestingly, the CY-derived MDSCs possessed immunosuppressive properties (15, 18). CY was reported to induce spleen colonization with an heterogenous myeloid population, including CD11b^+^Ly-6G^+^CD31^+^, which were found to inhibit proliferation of T cells (15, 24). In another study, conducted in tumor-bearing mice, CY was found to induce the production of CD11b^+^ Gr-1^lo^Ly6C^hi^CCR2^hi^ monocytic MDSCs (M-MDSCs) that suppress antitumor CD4^+^ effector cells through the PD-1/PD-L1 axis (25). Taken together, while lymphodepletion with CY may attenuate the risk of CRS it may also dampen the anti-cancer effects of CAR-T due to its preservation of Tregs and induction of MDSC.

## Effectiveness and safety of lymphodepletion with cyclophosphamide and fludarabine

The addition of Flu to CY appears to be beneficial, improving the expansion and the persistence of CAR-T-cells (6, 26). Moreover, FLU increased disease-free survival, rate and depth of response, OS and relapse-free survival (RFS) as well as the duration of B-cell aplasia compared to CY alone (6, 27–29). In fact, as evidenced by Jiang et al., absence of FLU in lymphodepletion as well as high lactate dehydrogenase (LDH) levels and low platelet counts pre-lymphodepletion were associated with higher relapse after CAR-T-cell therapy (30). Fludarabine is a purine analog prodrug that is rapidly converted in plasma to F-araA and accumulates in cells where it is phosphorylated to its active metabolite F-araATP by deoxycytidine kinase, an enzyme that is highly expressed in human lymphocytes (31). F-araA has a half-life of approximately 20 h *in vivo*, and its clearance depends on adequate renal function (32). In fact, creatinine clearance [CrCl(est)] below 80 ml/min was found to correlate with time to toxicity, independently of age (33). The main dose-limiting toxicities of FLU are those of other chemotherapy agents, namely myelosuppression and risk of infection. However, neurotoxicity is also of concern especially in patients with advanced disease or elderly patients with reduced renal function (34). Thus, these patients require careful monitoring and might need appropriate dose reduction. Somnolence and peripheral neuropathy occur frequently, immediately following fludarabine infusion, but are reversible and non-specific (35). Of particular importance are the late-onset (20–250 days) neurological symptoms such as progressive visual disturbances, peripheral neuropathy, ataxia, hemiparesis and dementia (34). Attention should be given not to confuse the latter with the CAR-T-cell-related immune effector cell associated neurotoxicity syndrome (ICANS), which usually occurs within 1 week following therapy, and is often associated with CRS (34). This highlights the importance of careful consideration of the fludarabine concentration used for lymphodepletion in patients receiving CD19-specific CAR-T-cell therapy. Fabrizio et al. found that the use of suboptimal fludarabine concentration (Area Under the Curve - AUC <13.8 mg × h/L) for lymphodepletion in patients with relapse-refractory (R/R) B-ALL significantly increased the risk of disease relapse however they cautioned that this should be weighed against the risk of development of toxicities with higher AUC doses (36). Analogous results were reported by Dekker et al. with the leukemia-free survival (LFS) and the duration of B cell aplasia being shorter in the FLU-underexposed (37). Similarly, Scordo et al. found that patients with aggressive B-cell NHL who received commercial axicabtagene ciloleucel (Axi-cel) an optimal AUC was associated with a lower risk of relapse/progression, however, a high AUC was associated with an increase incidence of any-grade ICANS (38). Unfortunately, the current method of FLU-dosing administration based on linear body surface area (BSA) has been found to result in variable medication exposure (39). FLU levels are not readily available in most institutions making FLU pharmacokinetics impractical for optimizing lymphodepletion whilst reducing relapse and ICANs. The ongoing global shortage of fludarabine has compelled the exploration of alternative lymphodepleting regimens (8).

## Introduction and assessment of bendamustine as a lymphodepletion strategy for CAR-T cell therapies

BEN is an alkylating agent that was designed to have both antimetabolite and alkylating properties (40). Apart from creating DNA interstrands, thus impairing DNA synthesis, it has also been found to induce apoptosis and inhibit mitotic checkpoints (41, 42). Another action that separates BEN from other alkylating agents is its activation of a base excision DNA repair pathway (42). It is metabolized by the liver and not excreted by the kidneys, which makes it ideal for patients with suppressed renal function. BEN has a short half-life of only 40 min, which is much shorter compared to that of CY and FLU (~ 8.88 h and ~ 10 h respectively) (32, 43, 44). Bone marrow suppression and particularly lymphopenia are frequently seen after BEN use, with CD4+ T cells being the most affected cell population (45). A significant body of literature has emerged recently, largely from the research efforts of our group, with a primary focus on the immunomodulatory properties of BEN (46). Our investigations have delved into BEN’s effects in the pre-allogeneic hematopoietic cell transplant (HCT) conditioning setting, and as a post-transplant therapy for GvHD, specifically targeting alloreactive T-cells and Tregs (47–55). PT-BEN exhibits a higher degree of lymphodepletion, which includes the elimination of Tregs, in contrast to PT-CY, and thus may also offer a distinct advantage over CY in the setting of pre-CAR-T cell lymphodepletion. We have reported that the suppressive effect of BEN in GvHD is partially due to its effects in expanding the CD11b+Gr-1+ myeloid cells (52). However, despite the BEN-induced upregulation of MDSCs, which may raise concerns about its use in lymphodepletion, we have seen an advantage in its ability to preserve GvL effects (54).

Although enhanced lymphodepletion intensity with CY-FLU may be associated with higher probability of a favorable cytokine profile (LDH, MCP-1 and IL-7), increased CAR-T expansion as well as efficacy and durability of CAR-T cell therapy, it may also increase the frequency of severe CRS and ICANs (56, 57). In fact, a post-lymphodepletion upregulation of MCP-1 and IL-7 and decrease in LDH are associated with increased probability of complete remission (58, 59). In the JULIET2 trial, BEN was used in some patients at 90 mg/m^2^ daily for 2 days for lymphodepletion prior to Tisagenlecleucel administration in adult patients with relapsed or refractory DLBCL. No difference in efficacy or safety was observed between patients that received BEN compared to CY-FLU (60). In another study, Ramos et al. showed that the addition of FLU 30 mg/m^2^/day to BEN 70 mg/m^2^/day given for 3 days, in patients with R/R Hodgkin lymphoma, increased the homeostatic cytokines IL-7 and IL-15 and was associated with higher CD30 CAR-T persistence and longer 1 year disease-free survival compared to BEN 90mg/m^2^/day for 2 days alone (61). Moreover, they found that, CRS was significantly higher in CY- based lymphodepletion compared to their BEN- based regimen with or without FLU (61). Similar results favoring the use of BEN over CY-FLU were reported by Ghilardi et al, in patients with relapsed or refractory DLBCL that received Tisagenlecleucel (62). Specifically, FLU/CY significantly reduced lymphocyte counts at the time of CAR-T infusion and was associated with higher post-lymphodepletion CRP and ferritin levels. Patients treated with CY-FLU also exhibited decreased hemoglobin levels and platelet counts when compared to those receiving BEN, resulting in a higher requirement for platelet and packed red blood cell transfusions following tisagenlecleucel infusion. Furthermore, patients in the CY-FLU group exhibited lower median neutrophil counts when compared to BEN and more Grade ≥3 neutropenia requiring more frequent administration of granulocyte colony-stimulating factor (GCSF). Conversely, patients who underwent BEN-based lymphodepletion experienced fewer occurrences of neutropenic fever, infections, and hospitalizations. BEN was also associated with reduced rates of any-grade CRS, any-grade ICANs, as well as severe ICANS when contrasted with CY-FLU. These differences were also linked to the more frequent usage of tocilizumab in CY-FLU recipients. When patients were stratified into groups with similar characteristics, it was observed that BEN-based lymphodepletion exhibited comparable efficacy to CY-FLU in terms of overall response rate and progression-free survival (PFS). Moreover, it resulted in prolonged OS. The investigators also drew the conclusion that there exists an optimal threshold level for lymphodepletion, beyond which further reduction in lymphocyte counts does not enhance CAR-T efficacy but may elevate the risk of toxicities (62). In another recent study, Garcia-Calvo et al. compared different lymphodepleting regimens as bridging therapies before either Axicabtagene Ciloleucel (axi-cel) or Tisagenlecleucel infusion in patients with R/R DLBCL (63). No difference in efficacy or safety was observed between the BEN and non-BEN-containing regimens in either CD19 CAR-T cell regimen. The rates of ICANS and CRS, all grades or grade ≥3 were similar as was non-relapse mortality. Moreover, peak CAR-T-cell expansion after infusion and area under the curve between day of infusion and day 28 were similar in BEN and non-BEN subgroups. While BEN did not provide an additional benefit in median PFS and OS, patients in the BEN subgroups were found to have a trend toward an increased complete response to CAR-T-cell therapy (63). Ahmed et al. assessed the feasibility and the efficacy of outpatient Tisagenlecleucel administration in adults with B-cell lymphoma with BEN being the most common outpatient and CY-FLU the most common inpatient lymphodepletion used. They found that outpatient lymphodepletion was associated with lower any-grade CRS and ICANS versus the inpatient while PFS and OS were similar in the two groups (64). Similar findings were observed by Ong et al. in patients with R/R aggressive B-cell lymphoma treated with Axicabtagene Ciloleucel (65). BEN was the most common lymphodepleting agent used in the outpatient setting resulting in comparable incidence of CRS, and severe ICANs, to CY-FLU, except for the occurrence of any grade ICANS, which was lower in the BEN group. In fact, patients that received BEN required lower dose of dexamethasone, supporting the finding that BEN lymphodepletion is associated with a reduced risk of neurotoxicity. Additionally, there were no significant differences in hazard ratios for disease progression, relapse, or mortality, or in the rates of severe infectious complications, despite observing higher rates of grade ≥3 neutropenia in CY-FLU-treated patients. Consequently, outpatient lymphodepletion using BEN, appears to be equally effective to CY-FLU and may represent a cost-effective, safe, and efficient alternative (65). In a recent investigation, Sidana et al. compared the two-day use of BEN 90 mg/m^2^/day with a three-day course of CY-FLU (300 mg/m^2^/day and 30 mg/m^2^/day respectively) before the administration of two different BCMA CAR-T cell regimens [ciltacabtagene autoleucel (cilta-cel) and idecabtagene vicleucel (ide-cel)] in patients with multiple myeloma. CAR-T cell expansion and lymphocyte recovery were found to be comparable between the lymphodepletion groups. However, both nadir absolute lymphocyte count (ALC) and ALC before the CAR-T cell infusion were found to be significantly lower in the CY-FLU group. The incidence of ICANS, CRS and ICU admissions were similar between the two regimens. Comparable rates of neutropenic fever, infections as well as use of G-CSF, IVIG, steroids, and tocilizumab were observed. Overall response rate (ORR) and PFS were not different, irrespective of the CAR-T cell product used. These investigators concluded that the use of BEN for lymphodepletion before BCMA CAR-T cell therapy demonstrates comparable safety and efficacy as CY-FLU (9). Due to the FLU shortage over the past year, we also adopted a single-agent lymphodepletion strategy using BEN at a dose of 90 mg/m² administered over two consecutive days prior to infusing commercial CAR-T products in all patients. Importantly, no unexpected adverse effects were observed in the fifteen patients treated in this manner.

## Summary

In summary, recent retrospective reports suggest that employing BEN lymphodepletion prior to CAR-T cell infusion may be associated with a reduction in adverse events, including CRS, ICANS, cytopenias and infections when compared to the use of CY-FLU. Importantly, this approach seems to maintain the effectiveness of CAR-T cell therapy. Additionally, BEN can be administered in an outpatient basis within a shorter timeframe, offering a cost-effective alternative. Its use has become particularly relevant during the recent period of nationwide FLU shortage. BEN’s unique immunomodulatory effects support its application as a lymphodepleting agent and merits a prospective comparison to CY-FLU through randomized clinical trials.

## Data availability statement

The original contributions presented in the study are included in the article/supplementary material. Further inquiries can be directed to the corresponding author.

## Author contributions

EK: Conceptualization, Investigation, Resources, Supervision, Writing – original draft, Writing – review and editing. DF: Conceptualization, Writing – original draft. MH: Writing – review and editing. SK: Writing – review and editing. RS: Writing – review and editing.
